# Editorial: PANoptosis and its role in T cell-based immunotherapy

**DOI:** 10.3389/fimmu.2025.1729986

**Published:** 2025-10-31

**Authors:** Lanyu Jing, Anze Yu

**Affiliations:** ^1^ Breast Department, Guangdong Provincial Hospital of Chinese Medicine, The Second Affiliated Hospital of Guangzhou University of Chinese Medicine, The Second Clinical College of Guangzhou University of Chinese Medicine, Guangdong Provincial Academy of Chinese Medical Sciences, Guangzhou, Guangdong, China; ^2^ Breast Disease Center, First Affiliated Hospital, Sun Yat-sen University, Guangzhou, Guangdong, China; ^3^ Department of Urology, First Affiliated Hospital, Sun Yat-sen University, Guangzhou, Guangdong, China

**Keywords:** PANoptosis, T cell, immunotherapy, cytokine - immunological terms, inflammation

PANoptosis, an integrated form of programmed cell death encompassing pyroptosis, apoptosis, and necroptosis, has emerged as a key mechanism at the intersection of inflammation and immunity (1). Acting through the PANoptosome complex, this multifaceted pathway not only regulates cellular homeostasis but also orchestrates immune activation and tissue remodeling. In the tumor immune microenvironment, PANoptosis plays dual roles: it can amplify anti-tumor immunity or, conversely, induce immune exhaustion and immune escape when dysregulated (2, 3). T cell-based immunotherapy—including immune checkpoint blockade (ICB) and chimeric antigen receptor (CAR) T cell therapy—has transformed the therapeutic landscape of multiple cancers (4). However, the variability in patient responses underscores the complexity of immune regulation and cell death signaling. Exploring how PANoptosis modulates T cell activity offers new insights into the mechanisms underlying immunotherapy resistance and tumor immune evasion. This Research Topic gathers a series of studies that collectively explore this dynamic interplay from molecular discovery to translational potential.

At the molecular level, Wang et al. applied a multi-omics strategy to classify renal cell carcinoma (RCC) into distinct metabolic subtypes. Their findings revealed that metabolic reprogramming tightly associates with PANoptosis-related signaling, defining prognostic subgroups and potential targets for precision immunotherapy. Building on the theme of molecular regulation, Tao et al. investigated cathepsin Z (CTSZ) in prostate cancer and found that its overexpression not only predicts poor clinical outcomes but also shapes an immunosuppressive microenvironment characterized by elevated PD-1 and PD-L1 expression—linking protease signaling to checkpoint regulation and PANoptotic control.

Expanding this exploration into other cancer types, Lei et al. developed a programmed cell death (PCD) score model for hepatocellular carcinoma (HCC) that integrates transcriptomic and single-cell analyses. Their identification of UBE2E1 as a key oncogenic driver associated with high PCD scores highlights how cell death dynamics affect T cell exhaustion and tumor aggressiveness. Complementing this, Wu et al. conducted a pan-cancer analysis of PLAG1 and demonstrated its functional role in promoting tumor proliferation and immune evasion in bladder cancer, further strengthening the connection between PANoptosis and immune modulation.

Moving from genomics to the tumor microenvironment, Liao et al. explored the clinical relevance of tumor mutation burden (TMB) across cancers and identified RPLP0 as a predictive biomarker for immunotherapy response. Interestingly, RPLP0 knockdown enhanced anti–PD-L1 efficacy in bladder cancer models, providing a tangible example of how PANoptosis-related translational control may affect therapeutic sensitivity. Similarly, Wan et al. synthesized current findings on immune cell PANoptosis in colorectal cancer, emphasizing that excessive immune cell death can undermine anti-tumor immunity and proposing strategies to restore immune balance through targeted interventions.

Beyond oncology, Chen et al. extended the concept of PANoptosis to diabetic retinopathy, identifying several hub genes that regulate inflammation and immune cell infiltration. Their integrative machine-learning approach underscores that PANoptotic signaling has broad implications in immune-related diseases beyond cancer. In parallel, Ma et al. reviewed advances in immunogenic cell death (ICD) and nanomaterial-based therapies, highlighting how nanotechnology can trigger PANoptosis-like mechanisms to promote antigen release and potentiate durable T cell immunity. These findings demonstrate that manipulating cell death pathways can synergize with T cell-based therapies to achieve improved clinical outcomes.

Together, the articles in this Research Topic illuminate PANoptosis as a pivotal immunoregulatory hub bridging cell death, metabolism, and immune activation. They reveal how fine-tuning PANoptotic signaling can shape tumor immunogenicity, determine therapeutic response, and open new avenues for combinatorial immunotherapy. Future studies integrating single-cell, spatial, and multi-omics technologies will be essential to define the spatial and temporal coordination of PANoptosis within the tumor microenvironment. By deepening our mechanistic understanding, researchers may unlock strategies to exploit PANoptosis for enhanced T cell function, reinvigorated anti-tumor immunity, and ultimately improved patient survival.

This Research Topic therefore not only consolidates current progress but also lays the foundation for translating PANoptosis biology into next-generation immunotherapeutic strategies.

## References

[1] Lin JF Wang TT Huang RZ. PANoptosis in cancer: bridging molecular mechanisms to therapeutic innovations. Cell Mol Immunol. (2025) 22:996–1011. doi: 10.1038/s41423-025-01329-z, PMID: 40721869 PMC12398562

[2] Zhang X Song S Huang Z. Z-DNA-binding protein 1 exacerbates myocardial ischemia–reperfusion injury by inducing noncanonical cardiomyocyte PANoptosis. Signal Transduct Target Ther. (2025) 10:333. doi: 10.1038/s41392-025-02430-5, PMID: 41052986 PMC12501347

[3] Zhang X Tang B Luo J. Cuproptosis, ferroptosis and PANoptosis in tumor immune microenvironment remodeling and immunotherapy: culprits or new hope. Mol Cancer. (2024) 23:255. doi: 10.1186/s12943-024-02130-8, PMID: 39543600 PMC11566504

[4] Rahim MK Okholm TLH Jones KB. Dynamic CD8(+) T cell responses to cancer immunotherapy in human regional lymph nodes are disrupted in metastatic lymph nodes. Cell. (2023) 186:1127–1143.e18. doi: 10.1016/j.cell.2023.02.021, PMID: 36931243 PMC10348701

